# Genomic, Epigenomic, Transcriptomic, Proteomic and Metabolomic Approaches in Atopic Dermatitis

**DOI:** 10.3390/cimb45060331

**Published:** 2023-06-20

**Authors:** Dalia Bratu, Daniel Boda, Constantin Caruntu

**Affiliations:** 1 Department of Dermatology, ‘Colentina’ Clinical Hospital, 020125 Bucharest, Romania; dalia_bratu@yahoo.com; 2 Department of Dermatology, ‘Carol Davila’ University of Medicine and Pharmacy, 050474 Bucharest, Romania; daniel.boda@yahoo.com; 3 Department of Dermatology, ‘Ponderas’ Academic Hospital, 014142 Bucharest, Romania; 4 Department of Dermatology, “Prof. N.C. Paulescu” National Institute of Diabetes, Nutrition and Metabolic Diseases, 011233 Bucharest, Romania; 5 Department of Physiology, “Carol Davila” University of Medicine and Pharmacy, 050474 Bucharest, Romania

**Keywords:** atopic dermatitis, genome, epigenome, transcriptome, proteome, metabolome

## Abstract

Atopic dermatitis (AD) is a chronic inflammatory skin disease with a high prevalence in the developed countries. It is associated with atopic and non-atopic diseases, and its close correlation with atopic comorbidities has been genetically demonstrated. One of the main roles of genetic studies is to comprehend the defects of the cutaneous barrier due to filaggrin deficit and epidermal spongiosis. Recently, epigenetic studies started to analyze the influence of the environmental factors on gene expression. The epigenome is considered to be a superior second code that controls the genome, which includes alterations of the chromatin. The epigenetic changes do not alter the genetic code, however, changes in the chromatin structure could activate or inhibit the transcription process of certain genes and consequently, the translation process of the new mRNA into a polypeptide chain. In-depth analysis of the transcriptomic, metabolomic and proteomic studies allow to unravel detailed mechanisms that cause AD. The extracellular space and lipid metabolism are associated with AD that is independent of the filaggrin expression. On the other hand, around 45 proteins are considered as the principal components in the atopic skin. Moreover, genetic studies based on the disrupted cutaneous barrier can lead to the development of new treatments targeting the cutaneous barrier or cutaneous inflammation. Unfortunately, at present, there are no target therapies that focus on the epigenetic process of AD. However, in the future, miR-143 could be an important objective for new therapies, as it targets the miR-335:SOX axis, thereby restoring the miR-335 expression, and repairing the cutaneous barrier defects.

## 1. Introduction

Atopic dermatitis (AD) is a chronic inflammatory skin disease with a high prevalence in the high-income countries [1,2,3,4,5]. Moreover, in low- and middle-income countries too, its prevalence has shown an increasing trend [6]. Both sexes are affected equally, with a peak observed in the first year of life [7,8,9,10]. Children have a prevalence between 15 and 25%; in some countries, such as the United States the prevalence is 30% [11]. On the other hand, adults have a prevalence between 1 and 10%, the presence of the disease being associated with the late onset or persistence from childhood [12,13,14,15,16,17,18,19,20,21,22,23]. Although AD can become symptomatic at any age, in 60% of the cases it develops in the first year of life, in 80–90% of the cases it develops until the age of 5 and only 26% of the adults develop the disease after 60 years of age. AD is considered to be an inherited and multifactorial disease [11,13,14,21,24].

The complex etiology of atopic dermatitis involving interactions between genetic predisposition, favoring and triggering factors causes skin barrier abnormalities and immune dysfunctions critical in the pathogenesis of the disease [7,11,12,25,26]. The positive history of atopic diseases is the biggest risk factor known in the development of AD [11,12,23]. The genetic factors increase the susceptibility for atopy but they do not always lead to clinical manifestations of the disease [11,12,25]. The discovery of the filaggrin gene in 2006 had a significant impact in the understanding of the atopic diseases in the subgroup related with filaggrin deficit [27,28]. AD is associated with other atopic diseases from the atopic march, such as allergic asthma, allergic rhinitis, and food allergies; it is also with non-atopic diseases [1,29,30,31,32,33]. The neuropsychiatric disorders have a major impact on patients’ life. Many people suffer from depression, and anxiety [33,34,35,36] and they have a higher risk of suicide [33,37,38], sleep deprivation, and reduced quality of life. The chronic psychological stress increases the symptoms of AD creating a vicious circle [33,39,40,41,42]. The serotonin metabolism is altered by the high level of proinflammatory cytokines, leading to higher levels of depression and anxiety [43,44,45,46].

Clinically, patients present with erythematous papules, plaques or vesicles, excoriated papules or plaques or chronic lichenified hyperpigmented lesions [47]. Usually, children have widespread polymorphic lesions that can involve any area of the body while adults present with circumscribed localized lesions usually on the arms, legs, hands, neck and the periorbital area, most of the time linearly distributed and lichenified. The lesions have chronic relapses and are associated with pruritus. According to Hanifin and Rajka, the diagnosis of AD requires the presence of at least three major criteria and three minor criteria. Over the years, other simplified diagnosis methods have been published [8,48,49,50,51,52]. Histopathologically, acute lesions present with moderate spongiosis, mild acanthosis and exocytosis of inflammatory cells. In subacute lesions, the degree of acanthosis increases. With time, the lesions develop scales and crusts above the thickened epidermis and dermal perivascular inflammatory infiltrate [53].

One of the main roles of genetic studies is to comprehend the defects of the cutaneous barrier [27]. The evolution of technology has allowed for a more precise analysis of the “nomes” (genome, epigenome, transcriptome, proteome, metabolome and phenome) for understanding the mechanisms that cause AD. Genetic studies based on the disrupted cutaneous barrier lead to the development of new treatments targeting the cutaneous barrier or cutaneous inflammation [54,55,56].

## 2. Etiology, Pathogenesis, Physiopathology

**Figure 1:**
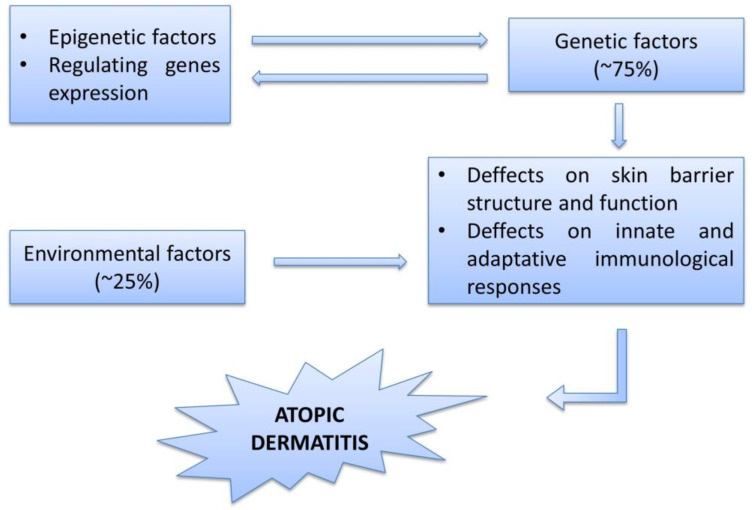
The etiology of atopic dermatitis. The complex etiology of the atopic dermatitis includes interactions between genetic predisposition, favoring and triggering factors. All these factors cause skin barrier abnormalities and immune dysfunctions critical in the pathogenesis of the disease. Adapted from Nedoszytko et al. 2020 [58].

The principal gene associated in several population with AD is the filaggrin gene, which is responsible for the integrity of the epithelium. Even if its mutations are a strong genetic risk factor in the European and Asian population, in African population their detection rate is very low. Moreover, investigations across different ethnicities are still limited [57]. Furthermore, the genetic factors increase the susceptibility for atopy, but they do not necessarily determine the clinical manifestations of the disease (Figure 1). The phenotype is the result of the complex interactions between the disrupted cutaneous barrier, the immunological responses and the environment. The skin barrier is very complex, with the capacity to maintain the equilibrium between the internal and the external environment. 

Patients with AD have a decreased bacterial diversity with decreased commensal flora but with significant Staphylococcus aureus; a higher bacterial diversity is observed after the treatment of the lesions or after their favorable evolution [59,60,61]. At the skin level, the quantity of Staphylococcus aureus is associated with the severity of the disease and with the number of relapses [62]. The complex pathophysiological processes associated with spongiosis and the consequences of filaggrin deficit lead to an excessive colonization due to a modified pH and also because the bacteria can migrate easily into the dermis. The migration causes cutaneous inflammation that leads to other cutaneous barrier imbalances [62,63,64,65,66,67]. In contrast, the intestinal microbiome influences AD in three ways: immunological, metabolic and neuroendocrine [68]. Probiotics interact in different ways with the intestinal epithelium, maintaining their balance. By modulating the release of various cytokines, they can induce the activation of the immune system signals, but can also induce the opposite effect, that is, tolerance [68,69,70,71]. Colonization of the skin with Staphylococcus aureus influences the severity and the number of relapses of AD, the relationship between them being directly proportional [62]. On the other hand, intestinal colonization can have a protective role in AD, as recent studies have demonstrated that it promotes the maturation of the immune system. There is little data about other Staphylococcus species, but several studies have concluded that a colonization of the skin with these species can have a positive impact in preventing AD by inhibiting the growth of Staphylococcus aureus [61,68,72]. On the other hand, a significant number of studies have demonstrated a high correlation between the lower amount of Malassezia, the high amount of filamentous fungi and also between Candida, Staphylococcus aureus and AD. The treatment targets to increase the amount of Malassezia in order to decrease the fungal colonization [73].

## 3. The Genome

**Figure 2:**
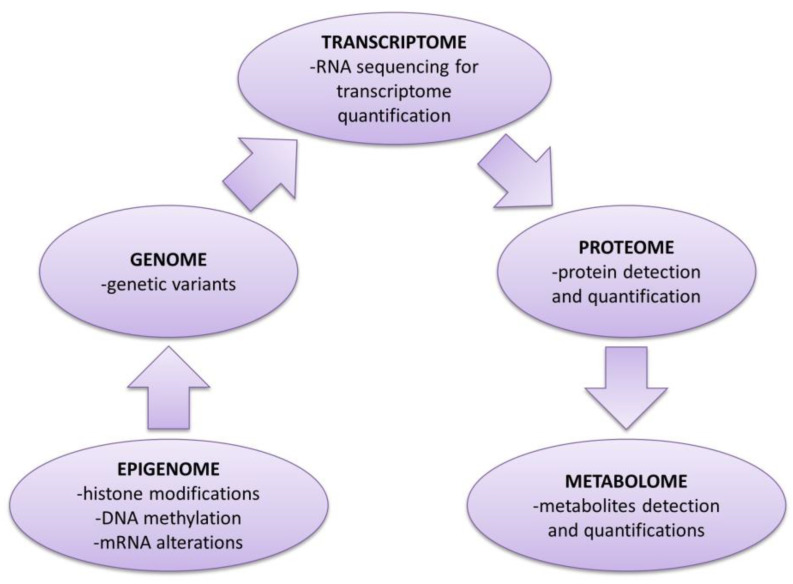
From genome to metabolome. The evolution of technology allowed for a more precise analysis of the “nomes” (genome, epigenome, transcriptome, proteome, metabolome and phenome) for understanding the mechanisms that cause the AD. Genetic studies based on the disrupted cutaneous barrier lead to the development of new treatments targeting the cutaneous barrier or cutaneous inflammation. Adapted with permission from Ritchie et al. 2015 [81].

Since the first genome-wide association studies (GWAS) on AD were published in the late 2000s, there were identified more than 30 loci, most of them associated with the development of the cutaneous barrier and immunologic dysfunctions. These studies are helpful in understanding the genetic risk [74,75,76]. On the other hand, the proteome-wide association study (PheWAS) reverse the research design of GWAS, analyzing more phenotypic variants associated with one genetic mutation. The purpose of the second type of study was to explain genetic pleiotropy [27,74,77]. Nowadays, it is possible to perform more extensive studies on rare mutations with the aid of whole-exome sequencing (WES) and whole-genome sequencing (WGS). WES analyzes the genetic sequencing of the exons DNA that encodes proteins and regions of the exons from the non-coding RNA. WES is rather helpful in studying rare diseases and in finding rare mutations that cause polygenic diseases. WGS sequences intergenic regions and exons because many of the regulatory mechanisms are situated in the intergenic regions of the DNA [27,78]. The mendelian randomization (MR) is useful in investigating the causal relationships. It uses the genetic variants to deduct the nongenetic variants caused by the environment. The genetic variants are already present before the onset of the disease, leading to less confusions while studying the risk factors (Figure 2) [74,79,80].

Studies on people from North America and Asia have shown that AD is associated with cardiometabolic diseases, obesity or overweight, hypertension, coronary artery disease, peripheral vascular disease, type II diabetes, etc. There is also a vicious circle among genetic factors, inflammation, sedentary lifestyle and corticotherapy, which can precipitate type II diabetes. In addition, due to pruritus, the quality of life decreases, sleep is affected, people becomes more sedentary, all of which increases the morbidity and the mortality of cardiovascular diseases [33,82,83,84,85,86,87]. For example, two potentially modifiable AD risk factors were analyzed using GWAS: vitamin D and obesity. The relationship between low vitamin D levels and AD resulted not to be a causal one [32,64], while the body mass index is highly associated with AD [32,65]. Further, modifying the risk factors associated with AD can decrease the incidence and the prevalence of the disease [74,82,88].

There are more than 70 genes associated with AD, and they are divided into five groups: genes leading to cutaneous barrier disfunction, genes associated with altered innate responses, genes associated with acquired immune responses, genes associated with stress response of the keratinocytes and genes involved in the vitamin D metabolism [58].

The filaggrin gene, located on chromosome 1q21.3, is responsible for the integrity of the cutaneous epithelium. Filaggrin is a structural and functional insoluble protein. Together with its precursor, profilaggrin and its degradation products, trans urocanic acid and pyrrolidone carboxylic acid, it contributes to the aggregation of intermediate filaments of keratin, inhibition of TEWL (transepidermal water loss), epidermal hydration, acidification, immunomodulation, and antibacterial effect. During keratinocyte differentiation, profilaggrin is dephosphorylated and degraded into monomers, which concentrate in the keratin cytoskeleton to form a large amount of protein–lipid matrix [59,89]. The null mutations of the gene are a major risk factor for AD but also for other atopic diseases, such as allergic asthma [28,58,90,91,92]. However, they show a certain population specificity, with important differences observed across various ethnicities. The frequency of the null mutations differs from country to country; around 10% of the Europeans are the carriers of a null mutation situated on exon 3 of the filaggrin gene [13,74,93]. It was explained by the natural immunization for infections which can be stimulated due to a permeable cutaneous barrier caused by the mutation [27,94].

A child’s risk of developing AD is 1.5 times higher if one of the parents has one atopic disease. The risk increases up to 3–5 times if one or both parents have AD. The concordance rate between monozygotic twins is 72–86%, while between dizygotic twins is 21–23%, which emphasizes the importance of the genetic component [58,91,92]. The carriers of two mutant alleles are almost always affected by AD, while patients with heterozygous mutations have an 8-fold risk of developing it [58,91]. Further, the number of filaggrin monomers that repeat in a sequence is responsible for the clinical phenotype and the onset and the severity of the disease [58,92].

**Figure 3:**
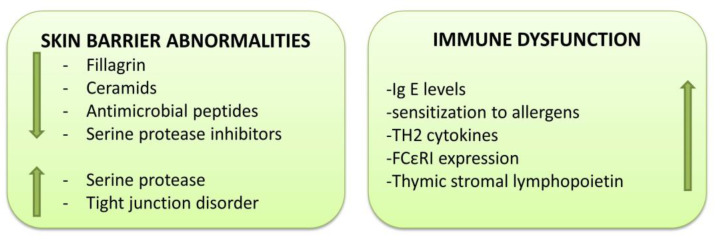
Skin barrier abnormalities and immune dysfunction in atopic dermatitis. The decrease in filaggrin, ceramides, antimicrobial peptides and serine protease inhibitors have a negative impact on AD, as the increase in serine proteases and the disorders of the tight junction affect the permeability of the cutaneous barrier. On the other hand, the immune dysfunction increases the risk of AD via the Th2 cells. Adapted from Yang et al. 2020 [113].

Besides the filaggrin gene, there are two other important genes. One leads to cutaneous barrier disfunction by regulating the filaggrin expression: ovo-like transcriptional repressor 1 (OLOV1), and the other one is associated with the innate immune response: interleukin 13 (IL-13) [58,95]. OLOV1 is a transcription factor that regulates filaggrin expression while IL-13 is responsible for type 2 T helper (Th2) cell responses [58]. The immune genes polymorphism is also associated with an increased risk of AD via the Th2 cells [59,96,97] since the Th2 responses predominate in AD. The Th2 responses decrease the production of cutaneous barrier proteins (filaggrin, loricrin, involucrin, cell adhesion proteins, desmosines, and claudins). Moreover, they impair the homeostasis of other epitheliums leading to an imbalanced immune response, which, in the end, is associated with systemic inflammation, airway hyperresponsiveness and alimentary allergies [59,89,98,99,100,101,102,103]. The IL-4 and IL-13 cytokines have an important role in producing chemokines, skin barrier disfunction due to lowering filaggrin expression, suppression of the antimicrobial peptides and allergic inflammation [59,98,104]. There are also other polymorphic genes involved in the development of AD, such as signal transducer and activator of transcription (STAT), thymic stromal lymphopoietin (TLSP), interferon regulatory factor 2, Toll-like receptor 2, high affinity Ig E receptor α (FcƐRI α), and vitamin D receptor; however, their importance is still under evaluation (Figure 3) [59,105,106,107,108,109,110,111,112].

All these genetic studies lead to the development of new treatments targeting the impaired cutaneous barrier and skin inflammation. Unfortunately, there are only a few systemic immunomodulatory biologic therapies approved for AD: Dupilumab (monoclonal antibody blocking IL-4 and IL-13) and Tralokilumab (human monoclonal antibody blocking IL-13). Lebrikizumab, another monoclonal antibody blocking IL-13, has the same mechanisms of action as Tralokilumab but it is still in phase three of development [54,55,56]. In the future, therapies could become more precise and also the adverse reactions and the costs for the treatments could decrease. Once the genetic risk is identified at birth, newborns could benefit from preventive treatments as daily emollients that prevent skin dryness and repair the skin barrier. Some studies state that AD prevention could decrease the risk of developing other comorbidities, such as asthma [74,114,115,116].

## 4. The Epigenome

The genetic studies demonstrated that the disease expresses in some carriers of mutations but at the same time, it also manifests in patients that do not carry any mutation [26,30,31]. Recently, the epigenetic studies started to analyze the influence of the environmental factors on gene expression [26]. The epigenome is considered a superior second code that controls the genome [26]. The epigenome includes alterations of the chromatin (covalent alterations of histone proteins, DNA methylations, and non-coding RNA-dependent regulations). These alterations can affect the DNA and the histone proteins, thereby modifying the gene expression in the genome [26]. The epigenetic changes do not directly modify the genetic code but changes in the chromatin structures could activate or inhibit the transcription of some genes and, moreover, the translation of the new mRNA [58,74,106,117,118,119,120,121,122,123,124,125,126,127,128].

The covalent alterations of histone proteins affect the chromatin compression level from lightly to tightly packed; the one lightly compressed becomes accessible for transcription [58,74,106,117,118,121]. The main epigenetic mechanism that has an impact on the gene expression regulation is the methylation of the cytosine, mostly where cytosine is followed by guanine. This mechanism blocks the transcription process, while the demethylation of cytosine stimulates the transcription [58,106,118,124,125,126]. Last but not least, the micro-RNAs are small non-coding proteins essential in regulating the posttranscriptional gene expression in the nucleated cells, as they are involved in many cellular processes. The micro-RNAs are involved in apoptosis, morphogenesis, proliferation, regulation of the cellular metabolism, signal transduction and cell differentiation [58,124,125,126,129,130]. The transcription of mi-RNA results in pri-mi-RNA, which is processed by the enzyme Drosha, generating pre-mi-RNA. Then, the mi-RNA leaves the nucleus and is processed in the cytoplasm by the DICER enzyme into a single-stranded mi-RNA. Drosha is a class 2 ribonuclease III enzyme, that in humans, is encoded by the DROSHA (formerly RNASEN) gene. It is the primary nuclease that executes the initiation step of miRNA processing in the nucleus [131]. When a micro-RNA binds to a specific mRNA it destabilizes it, leading to degradation in the cytoplasm and the removal of the mRNA. This process stops the translation and the gene function is inhibited. About 1–3% of the human genome could be regulated by epigenetic mechanisms [58,124,125,126,129,130].

Patients with AD have a different epigenetic profile due to variations in methylation profile and alterations in the expression of some specific micro-RNAs. The atopy was demonstrated to be imprinted in chromosomes 3, 6, 11, 13, and 14 [58,117,118,132,133,134,135,136]. Alterations in the expression of specific miRNAs are involved in regulating the expression of some genes that determine LTh2 polarization in the functionality of some LTreg, inflammatory processes, formation of tight junctions, proliferation and apoptosis of epidermal keratinocytes and also in the synthesis of cytokines and chemokines [133,135,137,138,139,140,141].

Some micro-RNAs were demonstrated to have more impact in the development of AD, and thus they could become the target of new possible treatments in the future. Ten species of miRNA are overexpressed in the atopic skin, while thirty-four species of miRNA are downregulated (Table 1) [58,117,118,132,133,134,135,136].

MiR-155 is overexpressed in patients with AD and it is essential for the differentiation of Th17 and regulatory T cells (Treg). Its overexpression is associated with sustained inflammation, severity of the disease and the percentage of Th17 lymphocytes [58,106,118,124,129,136,137,138,139,142,143,144]. The downregulation of the miR molecules from the Let-7-a-d family in patients with AD leads to the overproduction of IL-13 and CCR7 that promotes the predominance of Th2 responses. MiR-375 acts similarly, inducing the synthesis of thymic stromal lymphopoietin (TSLP), thereby blocking the expression of the transcription factor Kruppel-like *factor* 5 (KLF5) [58,129,138]. MiR-151a is involved in the pathogenesis of AD as it regulates the beta2 receptor of IL-12. It decreases the expression of the receptor when the molecule is stimulated. Along with hsa-mir-144-3p, it has been proposed as a possible biomarker in AD. The increased expression of hsa-mir-144-3p was observed in the umbilical cord fluid. On the other hand, the increased level of miR-151a was observed in the serum of AD patients [58,144]. MiR-29b has a positive correlation with SCORAD (tool for scoring the AD) [133]. MiR-143 inhibits IL-13, that when expressed, downregulates filaggrin, involucrin and loricrin [133]. MiR-335 and SOX work codependently: miR-335 is suppressed in patients with AD while SOX is upregulated. On the other hand, in healthy skin, the opposite phenomenon occurs. SOX6 is a transcription factor that suppresses epidermal differentiation by recruiting components of SWI/SNF-related matrix-associated actin-dependent regulator of chromatin subfamily A (SMARCA) complexes involved in keratinocyte differentiation. The loss of miR-335, together with the upregulation of SOX, affect the keratinocytes differentiation and cornification [145].

Facundes et al. tried to demonstrate whether IgG molecules from AD patients could regulate the functional properties of the thymic gamma-delta T cells (Tγδ), interact with the cell membranes of the Tγδ cells and also determine if they can control the miRNA expression. They concluded that the IgG molecules inhibit the expression of α4β7 integrin molecules, stimulate cutaneous lymphocyte-associated antigen (CLA) expression, interact with the Tγδ cell membrane through the receptors that are functionally differentiated by CTL activity and also interconnect with miR-181b-5p [146].

A lot of risk factors can alter the epigenome, from environment (pollution, and microbes) to therapy and supplement (probiotics, prebiotics, and folic acid), to pet allergens, obesity, stress, and smoking. The epigenetic changes during pregnancy could also affect the newborn, and then the offspring epigenome [58].

Smoking during pregnancy is associated with the early onset of atopic diseases in childhood [26]. Indirectly, it decreases Treg cells in the blood of the umbilical cord, which is associated with high risk of developing AD and alimentary allergies [26]. On the other hand, it increases the expression of miR-223 in the umbilical cord, which is associated with the decrease in Treg lymphocytes and an increased risk of developing AD under the age of three [26]. Furthermore, miR-223 inhibits the insulin growth factor 1 receptor (IGF1R) that has an important role in cellular metabolism, cellular proliferation and apoptosis [26]. The pollution decreases the expression of interferon gamma (IFNG) by methylation, leading to the development of Th2 dependent allergic reactions [26].

There is a positive association between the composition of commensal bacteria and the risk of developing allergies [58,147,148,149,150,151,152,153,154]. The children treated with antibiotics from an early age are at risk of developing allergies [26]. Moreover, the alteration of the function and the composition of the intestinal microbiome is involved in the pathogenesis of the metabolic diseases through pathways that involve covalent alterations of histone proteins, DNA methylations and non-coding RNA-dependent regulations [155,156,157]. A comparison between the methylation profile of the newborn blood cells from the umbilical cord of those living in rural areas and those living in urban areas revealed that in the blood cells of those living in urban areas, there was a decrease in IL-13 production with a lower activity of the Th2 cells, which is beneficial in allergy prevention [26]. Early exposure to microorganisms may influence the regulation of the gene expression, thus promoting a Th1 response [58,158,159,160,161,162].

A study found that the promoter gene Forkhead Box P3 (FOXP3) suffers demethylation in children whose mothers drink unpasteurized cow milk during pregnancy. Breastfeeding with unpasteurized cow milk leads to increased demethylation of the FOXP3 promoter gene in peripheral blood cells and increased Ltreg cells [58,158,159,160,161].

Simpson et al. performed a study on breast milk from 415 women, analyzing the miRNA profile, the influence of probiotics on the miRNA and the relationship between some changes in the miRNA profile and the development of AD in newborns. The expression of some miRNA differed between the placebo group and the group taking probiotics, however, further studies are still needed [130].

To date, there are no target therapies that focus on the epigenetic process of AD. In the future, MiR-143 could be an important target for new therapies. Furthermore, Liew et al. have recently demonstrated that Belinostat, whose target is the miR-335:SOX axis, restores miR-335 expression, thereby repairing the cutaneous barrier defects [133,145].

## 5. The Transcriptome, Proteome and Metabolome

The transcriptomic, metabolomic and proteomic analyses are quite laborious studies that help us better understand the pathological mechanisms of the disrupted skin barrier.

Cole et al. collected a high number of transcriptomes of atopic skin and demonstrated the importance of the extracellular space and lipidic metabolism [163]. Independent of the mutation type of the filaggrin gene, the carriers have an aberrant defense response. The study used the direct RNA sequencing for quantifying the entire transcriptome of the atopic skin of 26 patients. The conclusion was that even if seven genes that encode the extracellular region proteins are closely related to filaggrin expression, filaggrin expression does not modify the lipid composition of the horny layer. The expression of cartilage intermediate layer protein gene (CILP) leads to the most significant reduction in the expression of the null mutant filaggrin gene compared to the wild type. An overlap of the functional networks of proteins was identified that form the defense response through IFN 1. The amplification of this network can be associated with the response of the dysfunctional skin to viral infections in AD patients. However, this amplification could be a partially functional, suboptimal mechanism to compensate the high frequency of viral infections, including eczema herpeticum [163].

Using skin tape stripping, Goleva et al. identified around 45 proteins as the principal components in the anomalies of the atopic skin; most of them are expressed in patients with AD and food allergies [164]. The most important ones are the keratin intermediate filaments (KRT 5, KRT 8, KRT 10, KRT 14, KRT 16, KRT 17, and KRT 77), the proteins associated with inflammatory response (S100 calcium binding proteins, alarmins, and serine proteinase inhibitor glade B members), and the antioxidant and glycolytic enzymes. A high level of antioxidant and glycolytic enzymes prove the high energy need of those patients and also their need for defense with antioxidants at the skin barrier level [164]. All these anomalies are statistically correlated with the TEWL measured using the Tewameter device, along with total serum IgE (immunoglobulin E) and Staphylococcus aureus colonization [164,165,166,167]. The endotype of patients with AD and food allergies unfortunately persists into adulthood as well. TEWL allows for a non-invasive measurement of transcutaneous water loss. However, besides the impaired integrity of the cutaneous epithelium, TEWL can be influenced by skin thickness, skin temperature, anatomical site and also by the activity of the sweat glands [168,169,170]. Thus, its investigation may be crucial and it can explain the differences between various populations.

Transcutaneous water loss may precede the clinical manifestations of AD (eczema), so TEWL could be useful in the prevention of AD. Measurement of transcutaneous water loss in the first days of life could predict the development of AD in children independent of the filaggrin gene. The main proteins identified via skin tape stripping are positively associated with transcutaneous water loss and allergic sensitization [164,171].

There are numerous metabolites and metabolic pathways associated with atopic diseases since childhood, especially with allergic asthma. The easiest way is to analyze them from urine samples. The principal metabolites associated with AD are tryptophan, indoleacetic acid (increased in patients with AD and elevated total serum IgE), glycolic acid, taurocholic acid, taurochenodeoxycholic and glycochenodeoxycholic (decreased in patients with AD independent of the total serum IgE level), and cholic and chenodeoxycholic (increased in patients with AD and elevated total serum IgE) [172,173,174]. Other metabolites associated with AD are the acid 2-hydroxybutyrate (increased in patients with AD), hydroxyloctadecadienoic acids (increased in patients with AD and elevated total serum IgE), and sphingomyelins (altered levels in patients with AD and elevated total serum IgE) [172,173,174,175].

However, even if important progress has been achieved, investigations across different ethnic groups are still limited, and hence further studies on transcriptomes, proteomes, and metabolomes are needed for a more accurate understanding of these processes [163,164,172].

## 6. Conclusions

In the last two decades, the understanding of AD has significantly improved. The genetic studies based on the disrupted cutaneous barrier can lead to the development of immunomodulatory systemic biological therapies [26]. Further, DNA and RNA sequencing will substantially improve the prevention and the target therapies of the disease [54,55,56]. Along with other findings on genetic associations in AD and once the costs for genotyping becomes lower and the drugs become rather precise, the adverse reactions will decrease and the treatments will become less expensive [74]. On the other hand, preventing the risk factors of epigenetic changes, such as pollution, smoking, obesity, and stress, will decrease the incidence and prevalence of AD in the future. Moreover, the recent advances in the research of transcriptomes, proteomes and metabolomes, strengthen our confidence that they will have an important impact in unraveling the AD mechanisms, thereby opening new therapeutic pathways. Even though there are only a few systemic immunomodulatory biologic therapies approved for AD at present, in the future miRNAs could be an important target for new therapies.

## Figures and Tables

**Table 1:** MiRNA species related to AD.

**MiRNA Species**	**AD**	**References**
MiR-155	- overexpressed - essential for the differentiation of Th17 and Treg - associated with sustained inflammation, severity of the disease, and the percentage of Th17	[58,106,117,123,129,136,137,138,139,142,143,144]
Let-7-a-d-family	- downregulated - overproduction of IL-13 and CCR17 - predominance of Th2 responses	[58,129,138]
MiR-375	- similar with Let-7-a-d-family - induces the synthesis of TSLP - blocks the expression of KLF5	[58,129,138]
MiR-151a	- regulates the beta2 receptor of IL-12 - increased - biomarker AD	[58,143]
hsa-mir-144-3p	- biomarker AD - umbilical cord fluid	[58,143]
MiR-29b	- positive correlation with SCORAD	[133]
MiR-143	- inhibits IL-13 (when IL-13 is expressed, it downregulates filaggrin, involucrin and loricrin)	[133]
MiR-335 and SOX work codependently	- miR-335 is suppressed, while SOX is upregulated	[145]

Abbreviations: AD—atopic dermatitis; miR—microribonucleic acid; Th1—T helper cells 17; Treg—regulatory T cells; IL-13—interleukin 13; TSLP—thymic stromal lymphopoietin; KLF5—Kruppel-like *factor* 5; SCORAD—tool for scoring AD; SOX—a transcription factor.

## Data Availability

Not applicable.
